# Association of *EPAS1* and *PPARA* Gene Polymorphisms with High-Altitude Headache in Chinese Han Population

**DOI:** 10.1155/2020/1593068

**Published:** 2020-02-24

**Authors:** Yang Shen, Jihang Zhang, Jie Yang, Chuan Liu, Shizhu Bian, Chen Zhang, Jie Yu, Xubin Gao, Lan Huang

**Affiliations:** ^1^Institute of Cardiovascular Diseases of PLA, Xinqiao Hospital, Army Medical University, Chongqing, China; ^2^Department of Cardiology, Xinqiao Hospital, Army Medical University, Chongqing, China

## Abstract

**Background:**

High-altitude headache (HAH) is the most common complication after high-altitude exposure. Hypoxia-inducible factor- (HIF-) related genes have been confirmed to contribute to high-altitude acclimatization. We aim to investigate a possible association between HIF-related genes and HAH in the Chinese Han population.

**Methods:**

In total, 580 healthy Chinese Han volunteers were recruited in Chengdu (500 m) and carried to Lhasa (3700 m) by plane in 2 hours. HAH scores and basic physiological parameters were collected within 18–24 hours after the arrival. Thirty-five single nucleotide polymorphisms (SNPs) in HIF-related genes were genotyped, and linkage disequilibrium (LD) was evaluated by Haploview software. The functions of SNPs/haplotypes for HAH were developed by using logistic regression analysis.

**Results:**

In comparison with wild types, the rs4953354 “G” allele (*P*=0.013), rs6756667 “A” allele (*P*=0.013), rs6756667 “A” allele (*EPAS1*, and rs6520015 “C” allele in *PPARA* (*P*=0.013), rs6756667 “A” allele (*PPARA* (*P*=0.013), rs6756667 “A” allele (*EPAS1*, and rs6520015 “C” allele in *PPARA* (*P*=0.013), rs6756667 “A” allele (

**Conclusions:**

*EPAS1* and *PPARA* polymorphisms were associated with HAH in the Chinese Han population. Our findings pointed out potentially predictive gene markers, provided new insights into understanding pathogenesis, and may further provide prophylaxis and treatment strategies for HAH.*EPAS1*, and rs6520015 “C” allele in *PPARA* (

## 1. Introduction

High-altitude headache (HAH) is the most frequent complaint in lowlanders who ascend from plain area to high altitude and occurs either as an isolated symptom or as a part of acute mountain sickness (AMS) [1–4]. According to Lake Louise AMS scoring system revised in 2018, headache is an indispensable symptom [5]. HAH is defined by the International Headache Society as a disorder that typically develops within 24 hours after rapidly ascending to high altitude (≥2500 m) and resolves within 8 hours after descending [6]. Although HAH is not severe altitude sickness, it brings confusion, discomfort, and the poor state of emotional wellbeing and may even progress to life-threatening high-altitude cerebral edema (HACE) or high-altitude pulmonary edema (HAPE) [7]. About 80% of lowlanders are susceptible to HAH at altitudes higher than 3000 m; therefore, HAH has become a public health problem that demands prompt solution [4].

Multiple factors contribute to the development of HAH, including a history of headache, young age, female gender, obesity, strenuous exercise, dehydration, reaching altitude, and ascending speed, particularly at a speed of greater than 300–500 m/day [5, 8, 9]. With the increase of altitude, atmospheric pressure decreases which leads to a reduction of arterial oxygen pressure and activation of chemoreceptors. Basic pathophysiological changes include hypoxaemia, hyperventilation, and consequent cerebrovascular responses in the brain [10–12]. It is of vital importance to deliver sufficient oxygen to the brain via precise regulation of cerebral blood flow (CBF) to produce enough adenosine triphosphate and maintain the normal physiological function of brain tissue [13]. The increase of CBF elevates intracranial pressure, leading to brain swelling. Ross RT proposed the “tight-fit hypothesis” that people with a greater ratio of cranial cerebrospinal fluid to brain volume could compensate for the displacement of cerebrospinal fluid and be more tolerated to brain swelling and thus be less susceptible to HAH [14]. Yet another explanation emphasized that hypoxia caused cerebral edema and HAH by inducing damage of blood-brain barrier or by stimulating neurohumoral and hemodynamic responses, leading to cerebral vasodilation and overperfusion of microvascular beds via the release of inflammatory mediators [11, 15]. From previous studies of our group, Bian SZ and Guo WY reported that HAH patients featured higher vertebral artery diastolic velocity, higher heart rate (HR), higher self-rating anxiety scale score, and lower arterial oxygen saturation (SaO_2_) according to cohort study [16–18]. However, the exact pathophysiological mechanisms of HAH are multifactorial and far from specific elucidation.

Lack of oxygen is a common problem that people face at high altitude; thus, hypoxia is considered as the initial factor that triggers the development of HAH and AMS. However, lowlanders are more susceptible to high-altitude illness and are more likely to get brain function impairment than highlanders [19]. The concentration of blood hemoglobin, a typical indicator of hypoxia, is lower in Tibetans than in lowlanders when exposed to the same altitude [20]. Recently, genome-wide association studies have confirmed that genetic background differs between lowlanders and highlanders, which means that genomic loci have undergone natural selection in highlanders for hundreds of generations. Previous studies on the association between hypoxia adaptation and gene polymorphisms have focused on *hypoxia-inducible factor 1 alpha subunit* (*HIF-1A*, encoding HIF-1*α*), *hypoxia-inducible factor 1 alpha subunit inhibitor* (*HIF1AN*, encoding HIF-1*α* inhibitor), *egl-9 family hypoxia-inducible factor 1* (*EGLN1*, encoding prolyl hydroxylase domain-containing proteins-2, PHD2), *endothelial PAS domain protein 1* (*EPAS1*, encoding HIF-2*α*), and *peroxisome proliferator-activated receptor alpha* (*PPARA*, encoding peroxisome proliferator-activated receptor *α*, PPAR*α*) [21–27]. Family of HIF transcription factors play key roles in cellular and systemic adaptation including erythropoiesis, iron metabolism, vascular growth, and permeability [24]. Moreover, *EPAS1* has been approved to be associated with high-altitude adaptation in Tibetans and the occurrence of high-altitude illness including HAPE, HACE, and high-altitude polycythemia (HAPC) [28–30]. PPAR*α* is relevant to energy metabolism, specifically the fatty acid beta-oxidation in mitochondrial and peroxisomal under hypoxia conditions. The inhibition of PPAR*α* function may increase organs' susceptibility to oxidative damage [31, 32]. Furthermore, *HIF1A*, *HIF1AN*, and *EGLN1* participate in oxidative stress and the occurrence of metabolic diseases through HIF mediated transcriptional regulatory mechanism [21, 33–35]. So, we propose our hypothesis that associations may exist between HIF-related genetic factors and the susceptibility or resistance to HAH in the Chinese Han population.

For the above, the specific molecular mechanism of HAH remains unclear and the association between genetic variants and HAH after acute high-altitude exposure has been poorly understood. In the prospective cohort study, 35 SNPs in *EPAS1*, *EGLN1*, *HIF1A*, *HIF1AN*, and *PPARA* genes are selected, and the associations between SNPs and HAH in Chinese Han population are evaluated after rapidly ascending to Lhasa (3700 m) by plane. We aim to enhance the understanding of the concrete mechanisms of HAH, provide useful predictive information, and contribute to developing prevention and treatment strategies of HAH.

## 2. Materials and Methods

### 2.1. Study Population

The study enrolled 580 unrelated Chinese Han males aged from 18 to 45 years in Chengdu (500 m) in June 2012. All volunteers were military officers and soldiers, and detailed information including birthplace and permanent residence was collected. Han people who lived in the plain area permanently without high-altitude exposure history within 6 months were included. Subjects enrolled in the study took health examinations in Xinqiao Hospital (Chongqing, China) prior to the trip and would be excluded if they had a history of migraine or nonmigraine headache, cardiovascular diseases, respiratory diseases, neurological diseases, cerebral vascular diseases, cancer, and liver or kidney dysfunction. Individuals taking medicine or receiving prevention measures were also excluded.

### 2.2. Procedure and Data Collection

According to the study design, 580 participants were carried from Chengdu (500 m) to Lhasa (3700 m) by plane in 2 hours on June 29, 2012. Structured case report form questionnaires were designed to record basic demographic information including age, height, weight, body mass index (BMI), smoking/drinking status, and physiological parameters including HR, pulse oxygen saturation (SpO_2_), systolic blood pressure (SBP), and diastolic blood pressure (DBP). Distribution and completion of questionnaires were performed 7 days before the flight and within 18 to 24 hours after arrival at Lhasa (3700 m), respectively. HAH scores and physiological parameters were obtained in the next morning after arrival. HAH was diagnosed based on the criteria of International Classification of Headache Disorders: second edition, and the characteristics of headache need to meet at least two out of the five criteria: (1) bilateral, (2) frontal or frontotemporal, (3) dull or pressing pain, (4) mild to moderate intensity, and (5) aggravated by exertion, movement, straining, coughing, or bending down [4, 6]. Due to the difficult condition of field trial and clinical research, HAH scores were classified as follows: 0 for no headache, 1 for mild headache, 2 for moderate headache, and 3 for severe headache [16–18]. Individuals with HAH score ≥1 were assigned to the HAH group, while those with HAH score = 0 were classified into the non-HAH group. Physiological parameters were measured when participants had rested in a sitting position for more than 10 minutes. HR and BP were measured using a wrist sphygmomanometer (HEM-6200, OMRON Healthcare Ltd., Kyoto, Japan), while SpO_2_ was measured using a pulse oximeter (NONIN-9550, Nonin Onyx, Plymouth, MN, USA).

### 2.3. SNPs Selection and Genotyping

We selected 35 SNPs in *EPAS1*, *HIF1A*, *HIF1AN*, *EGLN1*, and *PPARA* from the dbSNP database (http://www.ncbi.nlm.nih.gov/projects/SNP/), and several of them had been reported to be associated with high-altitude illness or hypoxia in previous literatures [29, 30, 33]. SNPs that participated in adaptability adjustment of hypoxia located in noncoding regions more frequently than in coding regions, so we chose SNPs from introns, 3′ or 5′ untranslated regions, and upstream or downstream region of target genes. SNPs were excluded from further association analyses if minor allele frequency (MAF) was less than 5% or inconsistent with Hardy–Weinberg equilibrium (HWE).

Seven days before the trip, all subjects were requested to fast for 10–12 hours the night before, and we collected 5 ml peripheral venous blood by using K_2_-EDTA anticoagulant tubes from each subject on the next morning. After centrifugation, we extracted genomic DNA from blood cells by using the Ezup Column Blood Genomic DNA Extraction kit (Shanghai Sangon Biotechnology Co., Ltd., Shanghai, China) according to the manufacturer's instructions, and genomic DNA was stored at −20°C until analysis. We designed the polymerase chain reaction (PCR) primers with Sequenom MassARRAY Assay Design software (version 3.1; Sequenom, Inc., San Diego, CA, USA) and synthesized primers with Shanghai Sangon Biotechnology Co., Ltd (Shanghai, China). MALDI-TOF Mass Spectrometer (Sequenom, Inc., San Diego, CA, USA) was used for genotyping. Sequencing was performed in a blinded fashion, and 10% of DNA samples were repeatedly genotyped to guarantee the accuracy of results.

### 2.4. Statistical Analyses

All hypothesis testing analyses were two-sided, and a *P* value of <0.05 was considered as significant. Continuous variables were expressed as mean ± standard deviation, and differences between HAH group and non-HAH group were analyzed by independent samples *t*-test, while changes of physiological measurements after high-altitude exposure were analyzed by paired samples *t*-test. Categorical variables including smoking/drinking status were expressed as cases and percentages, and differences between groups were analyzed by the chi-square test. Allelic frequencies were calculated from the genotypes. Chi-square test was used to compare allele/genotype frequencies between groups and confirm that distributions of allele/genotype were in accordance with HWE. Binary logistic regression was used to analyze the association between variant genotypes and the risk of developing HAH under dominant and recessive models, and results were adjusted for potential confounders including age, BMI, smoking/drinking status, HR, SpO_2_, SBP, and DBP. Odds ratio (OR) and its approximate 95% confidence interval (CI) were calculated. Associations between gene variants and HAH intensity were analyzed using the Chi-squared test, by comparing mild HAH group and moderate-severe HAH group with the non-HAH group, respectively. Analyses of linkage disequilibrium (LD) and LD plot were performed by using Haploview 4.2 software. Microsoft Excel, SPSS 24.0 (SPSS Inc., Chicago, IL, USA), and SNPstats (https://www.snpstats.net) online software were also used for data processes and analyses.

## 3. Results

### 3.1. Characteristics of the Study Population

The study enrolled 580 healthy Chinese Han males, and the mean age was 23.04 ± 3.71 years. The characteristics of the study subjects are shown in Table 1. The incidence of HAH was 72.59%, and there were no cases of HACE or HAPE in this study. No significant difference was noted in the age, height, weight, BMI, drinking status, and baseline physiological parameters between the HAH group and the non-HAH group. However, smoking status significantly differed (51.07% in HAH group vs. 60.38% in non-HAH group, *P*=0.045). As for the physiological measurements at 3700 m, there were significantly higher HR (*P*=0.016) and lower SpO_2_ (*P*=0.007) in HAH patients. In general, subjects reported increased HR, SBP, DBP, and decreased SpO_2_ after high-altitude exposure (*P* < 0.001).

### 3.2. Associations between *EPAS1*/*PPARA* and HAH

Detailed information about target SNPs including allele, gene, chromosome position, MAF, HWE test, and genotypic distribution in 580 subjects is presented in Table 2. The results of the HWE test for all target SNPs were >0.05. In our study, rs4953354 and rs6756667 in *EPAS1* and rs6520015 in *PPARA* were found to be significantly associated with the risk of developing HAH.

Information of allelic distribution and association analyses between SNPs in *EPAS1* and HAH under multiple models is summarized in Table 3. The distribution of rs4953354 “A” allele (HAH, 89.0% vs. non-HAH, 83.0%) and “G” allele (HAH, 11.0% vs. non-HAH, 17.0%) was significantly different (*P*=0.007), implying that individuals carrying “G” allele were less susceptible to HAH compared with those who carry “A” allele. Under the codominant model, the genotypes “AG” (OR = 0.62; 95% CI = 0.41–0.95) and “GG” (OR = 0.25; 95% CI = 0.05–1.12) were associated with decreased HAH risk (*P*=0.023). Under the dominant model, the “AG/GG” genotype was significantly associated with decreased risk of HAH (OR = 0.59, 95% CI = 0.39–0.89, *P*=0.013) compared with the “AA” genotype. By comparing the allelic frequency of rs6756667 between two groups, we found that “A” allele (HAH, 10.0% vs. non-HAH, 15.1%) was significantly associated with decreased risk of HAH compared with “G” allele (HAH, 90.0% vs. non-HAH, 84.9%; *P*=0.014). Under the dominant model, the “AG/AA” genotype was significantly associated with decreased risk of HAH (OR = 0.63, 95% CI = 0.41–0.97, *P*=0.036) compared with the “AA” genotype. The results above remained significant after adjustment for multiple factors. However, no statistically significant association was found for rs4953354 or rs6756667 under the recessive model.

As summarized in Table 4, the distribution of rs6520015 “T” allele (HAH, 84.4% vs. non-HAH, 78.3%) and “C” allele (HAH, 15.6% vs. non-HAH, 21.7%, *P*=0.014) was significantly different, suggesting a dominant effect of “C” allele in lowering the HAH risk. Under the dominant model, the proportion of non-HAH subjects with at least one “C” allele was much higher than HAH patients (OR = 0.60, 95% CI = 0.41–0.88, *P*=0.009), and results remained significant after multivariable calibration. However, no association was found under the codominant model or recessive model. The “CA/AA” genotype of rs7292407 was associated with lower HAH risk (OR = 0.65, 95% CI = 0.43–0.98, *P*=0.041) compared with the “CC” genotype under dominant model; however, results turned insignificant after adjustment for multiple factors. No significant difference was found in the allelic distribution, under the codominant model or recessive model between the HAH group and non-HAH group.

No association was found between *HIF1A*/*HIF1AN*/*EGLN1* and HAH under the dominant model. Detailed information of association analyses between other SNPs and HAH under multiple models was summarized in Supplement [Supplementary-material supplementary-material-1].

### 3.3. Associations between *EPAS1*/*PPARA* and HAH Intensity

Subjects were divided into four groups according to HAH scores: 0 = non-HAH, 1 = mild HAH, 2 = moderate HAH, and 3 = severe HAH. The sample size of severe HAH was much smaller, so we combined it together with moderate HAH into one group when comparing allelic frequencies and genotypic distributions between groups. We found that rs4953354 and rs6520015 were associated with HAH intensity. As shown in Table 5, the distribution of rs4953354 “G” allele (mild HAH, 11.5% vs. non-HAH, 17.0%, *P*=0.017; moderate-severe HAH, 9.4% vs. non-HAH, 17.0%, *P*=0.023) was significantly different between groups. Under the dominant model, the “AG/GG” genotype of rs4953354 was significantly associated with decreased risk of mild HAH (mild HAH, 22.0% vs. non-HAH, 31.4%; *P*=0.024) and moderate-severe HAH (moderate-severe HAH, 18.8% vs. non-HAH, 31.4%; *P*=0.034). The distribution of rs6520015 “C” allele (mild HAH, 15.9% vs. non-HAH, 21.7%, *P*=0.026; moderate-severe HAH, 14.1% vs. non-HAH, 21.7%, *P*=0.042) was significantly different between groups. Under the dominant model, the “CT/CC” genotype of rs6520015 was significantly associated with decreased risk of mild HAH (mild HAH, 28.3% vs. non-HAH, 39.6%; *P*=0.011). No significant association was found between rs6520015 and moderate-severe HAH, and rs6756667 exhibited no significant difference in the development of mild HAH or moderate-severe HAH.

### 3.4. LD Analyses and Association between *PPARA* Haplotypes and HAH

**Figure 1:**
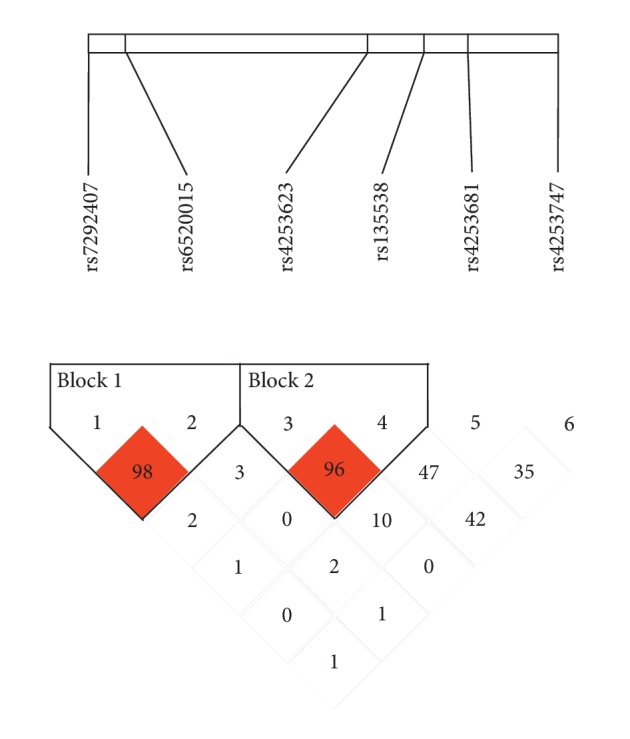
Haplotype block map for tag SNPs in *PPARA*.

The mutants of *EPAS1* and *PPARA* have been proved to be associated with HAH after acute high-altitude exposure. The loci of rs4953354 and rs6756667 located in the same gene, and the position of rs6520015 on the chromosome was close to rs7292407, rs4253623, rs135538, rs4253681, and rs4253747, so we drew linkage map of *EPAS1* and *PPARA*, respectively, using Haploview. As shown in Figure 1, two LD blocks were found in *PPARA*. Block 1 consisted of rs7292407 and rs6520015, and values of D′, *r*^2^, and LOD in Block 1 were 0.985, 0.812, and 121.37, respectively. Three common haplotypes (frequencies ≥ 1%) of Block 1 are exhibited in Table 6. Carriers of the rs7292407-rs6520015 “C-C” haplotype showed a lower risk of developing HAH (OR = 0.41, 95% CI = 0.19–0.89, *P*=0.024), and results remained significant after adjustment for multiple factors. Block 2 contained rs4253623 and rs135538, and values of D′, *r*^2^, and LOD in Block 2 were 0.966, 0.184, and 29.8, respectively (Figure 1). Three common haplotypes (frequencies ≥1%) of Block 2 were identified; however, no significant association was observed with HAH risk (Table 6). In addition, we found no haplotype block in *EPAS1* (Supplement [Supplementary-material supplementary-material-1]).

### 3.5. Combined Effects of *EPAS1* and *PPARA* with HAH Risk

Two loci (rs4953354 in *EPAS1* and rs6520015 in *PPARA*) were in association with HAH especially mild HAH after acute high-altitude exposure. We wondered whether subjects carrying two genes' mutants would show lower HAH risk compared with carriers of one mutant or no mutant at all. As rs6756667 played a weak role in the processes of HAH and made no difference in HAH intensity, we did not include it as a grouping criterion. Subjects were divided into 4 subgroups according to the carrying status of gene mutants (see Table 7). Compared with subjects in subgroup 1, subjects in subgroup 3 who carried rs6520015 “CT/CC” were less susceptible to HAH (OR = 0.61, 95% CI = 0.39–0.96, *P*=0.032); however, rs4953354 “AG/GG” carriers showed no significant difference. In the same way, subjects in subgroup 4 who carried both rs4953354 “AG/GG” and rs6520015 “CT/CC” performed larger proportion in the non-HAH group: 12.6% vs. 5.9% (OR = 0.35, 95% CI = 0.18–0.67, *P*=0.001) in comparison with subjects in subgroup 1. Results remained significant after multivariable calibration for age, BMI, smoking/drinking status, HR, SpO_2_, SBP, and DBP. In a word, *EPAS1* and *PPARA* gene mutants played a synergistic role in decreasing HAH risk after acute hypoxia exposure.

## 4. Discussion

To the best of our knowledge, we first established the association between *EPAS1* and *PPARA* polymorphisms and the risk of developing HAH in the Chinese Han population. Subjects carrying rs4953354 “AG/GG” genotype, rs6756667 “AG/AA” genotype, or rs6520015 “CT/CC” genotype were less susceptible to HAH compared with subjects carrying wild types. To be precise, rs4953354 was associated with decreased risk of mild HAH and moderate-severe HAH, whereas rs6520015 was associated with reducing the risk of mild HAH. LD phenomenon existed in *PPARA*, and rs7292407-rs6520015 “C-C” haplotype was associated with HAH. Individuals carrying both mutant type “AG/GG” genotype of rs4953354 and mutant type “CT/CC” genotype of rs6520015 showed a lower risk of HAH.

We detected a HAH incidence of 72.6% after high-altitude exposure in the current study, slightly lower than the incidence in previous studies [3, 4]. One possible reason is that subjects are general young soldiers, and they take exercise and military training regularly, resulting in better physical fitness and hypoxia tolerance. Differences in experiment design and ascending altitude have an impact as well. Total population exhibited significantly higher HR, SBP, DBP, and lower SpO_2_ before and after high-altitude exposure, and HAH patients showed significantly lower SpO_2_ and higher HR than non-HAH subjects, which was in accordance with previous researches [9]. To ensure sufficient oxygen supply, the activation of the autonomic nervous system and peripheral chemoreceptors may explain the changes of physiological parameters secondary to high-altitude exposure.

The *EPAS1* gene locates on the short arm of chromosome 2 between positions 21 and 16 and encodes the endothelial PAS domain protein 1 (EPAS1), which is an oxygen-sensitive alpha submit of HIF-2. Previous studies suggest that rs13419896, rs4953354, and rs6756667 in *EPAS1* are associated with high-altitude adaptation in Tibetans [21, 29, 32]. In our study, rs4953354 and rs6756667 are associated with decreased HAH risk in the Han population. The same genetic markers in both Tibetans and Han population confirmed the existence of an adaptive allele. However, unlike Han, Tibetans undergo natural selection for hundreds of generations, and the “G” allele on rs4953354 and “A” allele on rs6756667 have evolved into major alleles in *EPAS1* gene for the adaptation in the high-altitude region [27]. Our results also supported that those Han people who carry adaptive alleles may suffer less altitude sickness such as headache at high altitude. Under hypoxia conditions, a family of HIF transcription factors upregulate the expression level of genes involved in cellular and systemic hypoxia adaptation and play crucial roles in vascular responses [24, 36, 37]. Studies propose that EDN1, a vasoconstricting peptide primarily produced in vascular endothelium, could regulate vascular tone and take part in vascular homeostasis and migraine pathophysiology [38, 39]. What is more, EDN1 is regulated by HIF, for the expression of EDN1 has been observed to be reduced as the degradation of HIF-1*α* and HIF-2*α* [40]. Although underlying mechanisms are not determined, we deduce that *EPAS1* may contribute to HAH by regulating the expression of EDN1 through the HIF pathway. Moreover, rs4953354 and rs6756667 locate on the introns of *EPAS1*, in which the greatest genetic differences between Tibetans and the Chinese Han population exist [27]. Thus, we speculate that rs4953354 and rs6756667, close to the binding sites of several transcription factors of *EPAS1*, may alter the functions of transcription factors binding sites or be in linkage disequilibrium (LD) with other true functional SNPs, thus affecting *EPAS1* expression or regulating HIF relative genes in tissues experiencing hypoxia, and eventually assisting body adapting to the hypoxia condition in Chinese Han population. The rs13419896 may not directly participate in pathophysiological progress of oxygen metabolism and is irrelevant to HAH in our study. Further investigations should focus on finding more variants in or near *EPAS1* and explaining exact physiological mechanism associated with HAH.

The *PPARA* gene locates on chromosome 22 (46150521 bp–46243756 bp) and encodes peroxisome proliferator-activated receptor alpha (PPAR*α*). PPAR*α*, a member of nuclear receptor transcription factors family, is highly expressed in the heart, liver, kidney, and skeletal muscle where energy metabolism is quite active, regulating the lipid metabolism and gluconeogenesis, specifically the fatty acid beta-oxidation in mitochondrial and peroxisomal [41]. Hypoxia condition enhances the procedure of anaerobic glycolysis and lactic acid accumulation in venous blood that is regulated by PPAR*α* to enhance the utilization ratio of oxygen and keep the brain supplied with sufficient ATPs [42]. Decreased fatty acid oxidation in skeletal muscle is observed in Sherpa highlanders and Tibetans because fatty acid oxidation needs more oxygen when producing the same amount of ATPs [42, 43]. A putatively advantageous *PPARA* haplotype exhibited a significantly positive relationship with decreased expression or activity of PPAR*α* and increased serum free fatty acid in Tibetans, demonstrating that *PPARA* is associated with hypoxia metabolic adaptation [32]. Likewise, *PPARA* has an effect on vascular function, transcription of target genes, and decreased level of hemoglobin in Tibetans [44]. The rs6520015 “C” allele in *PPARA* shows a protective effect on the development of HAH in our study, which is different from the adaptation markers in Tibetans. The SNP locates on the introns of *PPARA* and is likely to be a noncoding variant. One possible explanation is that rs6520015 affects transcription regulation itself or may be in LD with a truly functional variant in a promoter or an enhancer element of *PPARA* and alter the expression level of *PPARA* as an expression quantitative trait locus. Under hypoxia conditions, microRNAs (miRNAs) are observed to be increased significantly in Tibetans than those in the Han population and strongly associated with red blood cell counts, hemoglobin concentration, and plasma concentration of erythropoietin [45]. miRNAs, a class of molecules which are approximate 22 nucleotides in length, belong to noncoding RNAs that regulate target gene expression at the posttranscriptional level. Moreover, *PPARA* is a potential target gene of miR-302b-5p. Combined with the phenomenon that rs7292407 and rs6520015 exhibit a strong LD and the haplotype “C-C” decreases HAH risk, although 2 SNPs locate 90 kb away from the transcription initiation site of *PPARA*, we extrapolate that the haplotype might change the structure of miRNA and further regulate the expression level of *PPARA* and its target genes, leading to the development of HAH at high altitude. According to our results, further investigations should focus on revealing the exact mechanism of how *PPARA* gene variants regulate the development of HAH.

The present study has several limitations. Firstly, 580 subjects enrolled in our research are Chinese Han young males and they are soldiers on military assignments. Age bias and sex bias may exist, and whether the results could be applied to all populations needs to be verified. Secondly, the diagnosis of HAH satisfied the criteria of the International Classification of Headache Disorders basically because it is difficult to record the detailed description of headache characteristics in a large field-based study. Subjects suffering HAH were not transferred to low-altitude immediately after the headache occurred because of military duty, but all HAH patients recovered after resting for four or five days at 3700 m with a follow-up observation. Lastly, exact mechanisms of how these SNPs affect genes function and pathophysiological process of HAH need to be verified by electrophoretic mobility shift assays and dual-luciferase reporter system in future studies.

## 5. Conclusion

We first demonstrated that rs4953354 and rs6756667 in *EPAS1* and rs6520015 in *PPARA* were significantly associated with decreased risk of HAH in the Chinese Han population. Our research examined the genetic characteristics of HAH and may evoke further investigation for underlying mechanisms involved in HAH etiology including functional characterization of transcription and expression. Findings also contributed to screening susceptible populations before ascending to high altitude and offering thinking for prevention and treatment strategies of HAH.

## Figures and Tables

**Table 1:** Basic characteristics of subjects.

Variables	Baseline measurements				Measurements at 3700 m				
	Total (580)	HAH+ (421)	HAH− (159)	*P* value^a^	Total (580)	HAH+ (421)	HAH− (159)	*P* value^a^	*P* value^#^
*Basic demographic data*									
Age (year)	23.04 ± 3.71	23.22 ± 3.89	22.58 ± 3.12	0.067	23.04 ± 3.71	23.22 ± 3.89	22.58 ± 3.12	0.067	—
Height (cm)	171.49 ± 4.64	171.42 ± 4.64	171.67 ± 4.64	0.571	171.49 ± 4.64	171.42 ± 4.64	171.67 ± 4.64	0.571	—
Weight (kg)	64.09 ± 7.42	64.19 ± 7.74	63.84 ± 6.50	0.615	64.09 ± 7.42	64.19 ± 7.74	63.84 ± 6.50	0.615	—
BMI (kg/m^2^)	21.78 ± 2.20	21.82 ± 2.29	21.65 ± 1.94	0.410	21.78 ± 2.20	21.82 ± 2.29	21.65 ± 1.94	0.410	—
Current smokers	311 (53.62)	215 (51.07)	96 (60.38)	0.045^b^^*∗*^	311 (53.62)	215 (51.07)	96 (60.38)	0.045^b^^*∗*^	—
Current drinkers	25 (4.31)	15 (3.56)	10 (6.29)	0.149^b^	25 (4.31)	15 (3.56)	10 (6.29)	0.149^b^	—

*Physiological parameters*									
HR (bpm)	65.17 ± 10.39	65.17 ± 10.63	65.17 ± 9.78	0.997	85.53 ± 12.97	86.33 ± 13.44	83.43 ± 11.42	0.016^*∗*^	<0.001^*∗*^
SpO_2_ (%)	98.14 ± 1.00	98.13 ± 1.02	98.19 ± 0.95	0.562	88.47 ± 3.14	88.25 ± 3.20	89.04 ± 2.91	0.007^*∗*^	<0.001^*∗*^
SBP (mmHg)	115.89 ± 10.83	115.91 ± 10.85	115.83 ± 10.82	0.945	118.39 ± 11.41	118.74 ± 11.85	117.45 ± 10.13	0.225	<0.001^*∗*^
DBP (mmHg)	73.81 ± 9.48	74.25 ± 9.76	72.64 ± 8.60	0.117	78.78 ± 9.83	79.23 ± 10.01	77.61 ± 9.26	0.077	<0.001^*∗*^

^a^Independent samples *t*-test. ^b^Chi-squared test (2^*∗*^ 2 contingency table, df = 1). ^*∗*^*P* < 0.05 indicated statistical significance. ^#^Comparison of physiological parameters between baseline and measurements at 3700 m in all subjects. HAH+, subjects with HAH; HAH−, subjects without HAH. HAH, high-altitude headache; BMI, body mass index; HR, heart rate; SpO_2_, pulse oxygen saturation; SBP, systolic blood pressure; DBP, diastolic blood pressure.

**Table 2:** SNP information and genotype distribution in 580 subjects.

SNP	Allele	Gene	Chromosome position	*N*	Genotype frequencies	MAF (%)	*P* value for HWE test
rs13419896	G/A	*EPAS1*	Chr2:46329206	577	0.47/0.43/0.10	31.0	1.000
rs4953354	A/G	*EPAS1*	Chr2:46348249	580	0.76/0.23/0.01	13.0	0.460
rs6756667	G/A	*EPAS1*	Chr2:46352270	580	0.78/0.21/0.01	11.0	0.680
rs7292407	C/A	*PPARA*	Chr22:46057832	549	0.74/0.23/0.03	15.0	0.090
rs6520015	T/C	*PPARA*	Chr22:46067551	580	0.69/0.28/0.03	17.0	0.770
rs4253623	A/G	*PPARA*	Chr22:46154203	575	0.75/0.23/0.02	13.0	1.000
rs135538	G/C	*PPARA*	Chr22:46168728	577	0.33/0.46/0.21	44.0	0.180
rs4253681	T/C	*PPARA*	Chr22:46183703	579	0.64/0.32/0.04	20.0	0.890
rs4253747	T/A	*PPARA*	Chr22:46217340	580	0.63/0.33/0.04	27.0	0.900
rs2009873	A/G	*EGLN1*	Chr1:231363490	576	0.34/0.48/0.18	42.0	0.550
rs2066140	G/C	*EGLN1*	Chr1:231368565	578	0.34/0.48/0.18	42.0	0.800
rs2739513	A/G	*EGLN1*	Chr1:231379455	563	0.34/0.48/0.18	42.0	0.600
rs2486736	A/G	*EGLN1*	Chr1:231385732	572	0.33/0.49/0.18	42.0	1.000
rs480902	C/T	*EGLN1*	Chr1:231395881	579	0.34/0.48/0.18	42.0	0.860
rs508618	A/G	*EGLN1*	Chr1:231396566	578	0.78/0.21/0.01	11.0	0.130
rs2790882	A/G	*EGLN1*	Chr1:231397037	575	0.33/0.49/0.18	43.0	0.930
rs2486729	A/G	*EGLN1*	Chr1:231399838	576	0.34/0.45/0.20	43.0	0.089
rs7542797	A/C	*EGLN1*	Chr1:231418041	577	0.76/0.22/0.01	12.0	0.850
rs1339891	G/A	*EGLN1*	Chr1:231420649	579	0.81/0.18/0.01	10.0	1.000
rs12406290	A/G	*EGLN1*	Chr1:231423480	561	0.28/0.49/0.22	47.0	0.870
rs2153364	A/G	*EGLN1*	Chr1:231424474	530	0.28/0.49/0.23	48.0	0.073
rs2275279	A/T	*EGLN1*	Chr1:231591348	579	0.54/0.38/0.08	27.0	0.250
rs2301104	G/C	*HIF1A*	Chr14:61698310	579	0.87/0.13/0.00	7.0	0.500
rs12434438	A/G	*HIF1A*	Chr14:61730580	568	0.56/0.39/0.05	24.0	0.170
rs966824	C/T	*HIF1A*	Chr14:61733800	574	0.68/0.30/0.02	17.0	0.380
rs2301112	A/C	*HIF1A*	Chr14:61739455	545	0.91/0.09/0.00	5.0	1.000
rs2301113	A/C	*HIF1A*	Chr14:61739830	576	0.43/0.46/0.10	34.0	0.400
rs2295778	C/G	*HIF1AN*	Chr10:100536079	578	0.58/0.36/0.06	24.0	0.910
rs11190602	T/C	*HIF1AN*	Chr10:100537499	578	0.78/0.20/0.02	12.0	0.420
rs3750633	G/A	*HIF1AN*	Chr10:100548457	579	0.85/0.14/0.01	8.0	0.390
rs10883512	A/G	*HIF1AN*	Chr10:100548759	579	0.85/0.14/0.01	8.0	0.560
rs11816840	G/C	*HIF1AN*	Chr10:100549463	579	0.85/0.14/0.01	8.0	0.770
rs1054399	C/T	*HIF1AN*	Chr10:100552808	579	0.85/0.14/0.01	8.0	0.390
rs11292	T/C	*HIF1AN*	Chr10:100553850	580	0.85/0.14/0.01	8.0	0.390
rs11190613	T/C	*HIF1AN*	Chr10:100554240	580	0.85/0.14/0.01	8.0	0.390

SNP, single nucleotide polymorphism; MAF, minor allele frequency; HWE, Hardy–Weinberg equilibrium.

**Table 3:** Association between SNPs in *EPAS1* and HAH under multiple genetic models.

SNP	Model	Allele/genotype	HAH+ [*n* (%)]	HAH− [*n* (%)]	OR (95% CI)	*P* value	OR (95% CI)^a^	*P* value^a^
rs4953354	Allele	A	749 (89.0)	264 (83.0)		0.007^*∗*^		
		G	93 (11.0)	54 (17.0)				
	Codominant	AA	331 (78.6)	109 (68.5)	1	0.023^*∗*^	1	0.046^*∗*^
		AG	87 (20.7)	46 (28.9)	0.62 (0.41–0.95)		0.61 (0.40–0.94)	
		GG	3 (0.7)	4 (2.5)	0.25 (0.05–1.12)		0.37 (0.08–1.74)	
	Dominant	AA	331 (78.6)	109 (68.5)	1	0.013^*∗*^	1	0.016^*∗*^
		AG/GG	90 (21.4)	50 (31.4)	0.59 (0.39–0.89)		0.59 (0.39–0.90)	
	Recessive	AA/AG	418 (99.3)	155 (97.5)	1	0.097	1	0.250
		GG	3 (0.7)	4 (2.5)	0.28 (0.06–1.26)		0.41 (0.09–1.93)	

rs6756667	Allele	G	758 (90.0)	270 (84.9)		0.014^*∗*^		
		A	84 (10.0)	48 (15.1)				
	Codominant	GG	339 (80.5)	115 (72.3)	1	0.030^*∗*^	1	0.061
		AG	80 (19)	40 (25.2)	0.68 (0.44–1.05)		0.67 (0.43–1.05)	
		AA	2 (0.5)	4 (2.5)	0.17 (0.03–0.94)		0.23 (0.04–1.29)	
	Dominant	GG	339 (80.5)	115 (72.3)	1	0.036^*∗*^	1	0.044^*∗*^
		AG/AA	82 (19.5)	44 (27.7)	0.63 (0.41–0.97)		0.64 (0.41–0.98)	
	Recessive	GG/AG	419 (99.5)	155 (97.5)	1	0.044	1	0.100
		AA	2 (0.5)	4 (2.5)	0.18 (0.03–1.02)		0.25 (0.04–1.41)	

Allelic frequencies were compared by the Chi-squared test (2 ^*∗*^2 contingency table, df = 1). Association between SNPs and HAH under different models was detected by using binary logistic regression. ^a^Adjusted for age, height, weight, BMI, smoking and drinking status, HR, SpO_2_, SBP, and DBP. ^*∗*^*P* < 0.05 indicated statistical significance. HAH+, subjects with HAH; HAH−, subjects without HAH. OR, odds ratio; 95% CI, 95% confidence interval; SNP, single nucleotide polymorphism; HAH, high-altitude headache; BMI, body mass index; HR, heart rate; SpO_2_, pulse oxygen saturation; SBP, systolic blood pressure; DBP, diastolic blood pressure.

**Table 4:** Association between SNPs in *PPARA* and HAH under multiple genetic models.

SNP	Model	Allele/genotype	HAH+ [*n* (%)]	HAH− [*n* (%)]	OR (95% CI)	*P* value	OR (95% CI)^a^	*P* value^a^
rs7292407	Allele	C	685 (86.5)	251 (82.0)		0.061		
		A	107 (13.5)	55 (18.0)				
	Codominant	CC	301 (76.0)	103 (67.3)	1	0.110	1	0.150
		CA	83 (21.0)	45 (29.4)	0.63 (0.41–0.97)		0.64 (0.42–1.00)	
		AA	12 (3.0)	5 (3.3)	0.82 (0.28–2.39)		0.93 (0.31–2.79)	
	Dominant	CC	301 (76.0)	103 (67.3)	1	0.041^*∗*^	1	0.066
		CA/AA	95 (24.0)	50 (32.7)	0.65 (0.43–0.98)		0.67 (0.44–1.02)	
	Recessive	CC/CA	384 (97.0)	148 (96.7)	1	0.890	1	0.930
		AA	12 (3.0)	5 (3.3)	0.93 (0.32–2.67)		1.05 (0.35–3.11)	

rs6520015	Allele	T	711 (84.4)	249 (78.3)		0.014^*∗*^		
		C	131 (15.6)	69 (21.7)				
	Codominant	TT	302 (71.7)	96 (60.4)	1	0.034^*∗*^	1	0.059
		CT	107 (25.4)	57 (35.9)	0.60 (0.40–0.89)		0.61 (0.41–0.92)	
		CC	12 (2.8)	6 (3.8)	0.64 (0.23–1.74)		0.72 (0.25–2.02)	
	Dominant	TT	302 (71.7)	96 (60.4)	1	0.009^*∗*^	1	0.018^*∗*^
		CT/CC	119 (28.3)	63 (39.6)	0.60 (0.41–0.88)		0.62 (0.42–0.92)	
	Recessive	TT/CT	409 (97.2)	153 (96.2)	1	0.570	1	0.750
		CC	12 (2.8)	6 (3.8)	0.75 (0.28–2.03)		0.84 (0.30–2.35)	

Allelic frequencies were compared by Chi-squared test (2^*∗*^ 2 contingency table, df = 1). Association between SNPs and HAH under different models was detected by using binary logistic regression. ^a^Adjusted for age, height, weight, BMI, smoking and drinking status, HR, SpO_2_, SBP, and DBP. ^*∗*^*P* < 0.05 indicated statistical significance. HAH+, subjects with HAH; HAH−, subjects without HAH. See Table 3 for group abbreviations.

**Table 5:** Association between rs4953354, rs6756667, rs6520015, and HAH intensity.

SNP	Model	Allele/genotype	non-HAH [*n* (%)]	Mild HAH [*n* (%)]	Moderate-severe HAH [*n* (%)]	*P* value^a^	*P* value^b^
rs4953354	Allele	A	264 (83.0)	595 (88.5)	154 (90.6)	0.017^*∗*^	0.023^*∗*^
		G	54 (17.0)	77 (11.5)	16 (9.4)		
	Dominant	AA	109 (68.6)	262 (78.0)	69 (81.2)	0.024^*∗*^	0.034^*∗*^
		AG/GG	50 (31.4)	74 (22.0)	16 (18.8)		
	Recessive	AA/AG	155 (97.5)	333 (99.1)	85 (100.0)	0.219	0.301
		GG	4 (2.5)	3 (0.9)	0 (0.0)		

rs6756667	Allele	G	270 (84.9)	603 (89.7)	155 (91.2)	0.028^*∗*^	0.049^*∗*^
		A	48 (15.1)	69 (10.3)	15 (8.8)		
	Dominant	GG	115 (72.3)	269 (80.1)	70 (82.4)	0.054	0.081
		AG/AA	44 (27.7)	67 (19.9)	15 (17.6)		
	Recessive	GG/AG	155 (97.5)	334 (99.4)	85 (100.0)	0.087	0.301
		AA	4 (2.5)	2 (0.6)	0 (0.0)		

rs6520015	Allele	T	249 (78.3)	565 (84.1)	146 (85.9)	0.026^*∗*^	0.042^*∗*^
		C	69 (21.7)	107 (15.9)	24 (14.1)		
	Dominant	TT	96 (60.4)	241 (71.7)	61 (71.8)	0.011^*∗*^	0.077
		CT/CC	63 (39.6)	95 (28.3)	24 (28.2)		
	Recessive	TT/CT	153 (96.2)	324 (96.4)	85 (100.0)	0.911	0.095
		CC	6 (3.8)	12 (3.6)	0 (0.0)		

Association between SNPs and HAH intensity was analyzed in allele-dose, under dominant and recessive model by using Chi-squared test (2 ^*∗*^2 contingency table, df = 1). ^a^Mild HAH group vs. non-HAH group; ^b^Moderate-severe HAH group vs. non-HAH group. ^*∗*^*P* < 0.05 indicated statistical significance. See Table 3 for group abbreviations.

**Table 6:** Distributions of *PPARA* haplotypes and the association with HAH risk.

Haplotypes		Frequencies		OR (95% CI)	*P* value	OR (95% CI)^a^	*P* value^a^
		HAH+	HAH−				
rs7292407	rs6520015						
C	T	0.783	0.826	1		1	
A	C	0.175	0.147	0.71 (0.50–1.01)	0.057	0.74 (0.52–1.06)	0.110
C	C	0.042	0.026	0.41 (0.19–0.89)	0.024^*∗*^	0.41 (0.19–0.92)	0.030^*∗*^
Global haplotype association *P* value: 0.045^*∗*^							

rs4253623	rs135538						
A	G	0.552	0.565	1		1	
A	C	0.316	0.293	1.09 (0.83–1.45)	0.530	1.14 (0.86–1.53)	0.370
G	C	0.131	0.137	0.97 (0.66–1.45)	0.900	1.02 (0.68–1.53)	0.940
Global haplotype association *P* value: 0.710							

Associations between *PPARA* haplotypes and HAH risk were detected by binary logistic regression. ^a^Adjusted for age, height, weight, BMI, smoking and drinking status, HR, SpO_2_, SBP, and DBP. ^*∗*^*P* < 0.05 indicated statistical significance. HAH+, subjects with HAH; HAH−, subjects without HAH. See Table 3 for group abbreviations.

**Table 7:** Distribution of rs4953354 and rs6520015 in HAH and non-HAH groups.

Subgroups	HAH+ [*n* (%)]	HAH− [*n* (%)]	OR (95% CI)	*P* value	OR (95% CI)^a^	*P* value^a^
1	237 (56.29)	66 (41.51)	1		1	
2	65 (15.44)	30 (18.87)	0.60 (0.36–1.01)	0.053	0.60 (0.35–1.02)	0.059
3	94 (22.33)	43 (27.04)	0.61 (0.39–0.96)	0.032^*∗*^	0.63 (0.40–0.99)	0.049^*∗*^
4	25 (5.94)	20 (12.58)	0.35 (0.18–0.67)	0.001^*∗*^	0.36 (0.18–0.70)	0.003^*∗*^

Subgroup 1: subjects carrying neither rs4953354 “AG/GG” nor rs6520015 “CT/CC”; subgroup 2: subjects carrying rs4953354 “AG/GG” but not rs6520015 “CT/CC”; subgroup 3: subjects carrying rs6520015 “CT/CC” but not rs4953354 “AG/GG”; subgroup 4: subjects carrying both rs4953354 “AG/GG” and rs6520015 “CT/CC”. ^a^Adjusted for age, height, weight, BMI, smoking and drinking status, HR, SpO_2_, SBP, and DBP. ^*∗*^*P* < 0.05 indicated statistical significance. HAH+, subjects with HAH; HAH−, subjects without HAH. See Table 3 for group abbreviations.

## Data Availability

The data used to support the findings of this study are available from the corresponding author upon request.

## Supplementary Materials

Supplementary Materials Supplement Table 1: association between other SNPs and HAH under multiple models. Figure S1: haplotype block map for tag SNPs in EPAS1.
