# Supplementary material for: Association of EPAS1 and PPARA Gene Polymorphisms with High-Altitude Headache in Chinese Han Population
Source: Biomed Res Int. 2020 Feb 24;2020:1593068. doi: 10.1155/2020/1593068 (PMC7060407; doi:10.1155/2020/1593068)
Supplement: Supplementary Materials — Supplement Table 1: association between other SNPs and HAH under multiple models. Figure S1: haplotype block map for tag SNPs in EPAS1. [file 1593068.f1.zip › 1593068.f1/Final Supplement Figure 1.docx]

rs6756667

rs4953354

rs13419896

1

3

2

41

2

1

Figure S1: Haplotype block map for tag SNPs in EPAS1
