# Supplementary material for: Association of EPAS1 and PPARA Gene Polymorphisms with High-Altitude Headache in Chinese Han Population
Source: Biomed Res Int. 2020 Feb 24;2020:1593068. doi: 10.1155/2020/1593068 (PMC7060407; doi:10.1155/2020/1593068)
Supplement: Supplementary Materials — Supplement Table 1: association between other SNPs and HAH under multiple models. Figure S1: haplotype block map for tag SNPs in EPAS1. [file 1593068.f1.zip › 1593068.f1/Final Supplement Table 1.docx]

Table S1:Association between other SNPs and HAH under multiple models.

| **SNP** | **Model** | **Allele/Genotype** | **HAH+ [n (%)]** | **HAH- [n (%)]** | **OR (95% CI)** | **P value** | **OR (95% CI)^a^** | **P value^a^** |
| --- | --- | --- | --- | --- | --- | --- | --- | --- |
| rs13419896 | Allele | G | 574 (68.5) | 217 (68.7) |  | 0.955 |  |  |
|  |  | A | 264 (31.5) | 99 (31.3) |  |  |  |  |
|  | Codominant | GG | 196 (46.8) | 75 (47.5) | 1 | 0.970 | 1 | 0.880 |
|  |  | AG | 182 (43.4) | 67 (42.4) | 1.04 (0.71-1.53) |  | 1.01 (0.68-1.51) |  |
|  |  | AA | 41 (9.8) | 16 (10.1) | 0.98 (0.52-1.85) |  | 1.18 (0.61-2.29) |  |
|  | Dominant | GG | 196 (46.8) | 75 (47.5) | 1 | 0.880 | 1 | 0.820 |
|  |  | AG/AA | 223 (53.2) | 83 (52.5) | 1.03 (0.71-1.48) |  | 1.05 (0.72-1.53) |  |
|  | Recessive | GG/AG | 378 (90.2) | 142 (89.9) | 1 | 0.900 | 1 | 0.620 |
|  |  | AA | 41 (9.8) | 16 (10.1) | 0.96 (0.52-1.77) |  | 1.17 (0.62-2.21) |  |
| rs4253623 | Allele | A | 724 (86.8) | 271 (85.8) |  | 0.641 |  |  |
|  |  | G | 110 (13.2) | 45 (14.2) |  |  |  |  |
|  | Codominant | AA | 316 (75.8) | 114 (72.2) | 1 | 0.190 | 1 | 0.160 |
|  |  | AG | 92 (22.1) | 43 (27.2) | 0.77 (0.51-1.18) |  | 0.77 (0.50-1.20) |  |
|  |  | GG | 9 (2.2) | 1 (0.6) | 3.25 (0.41-25.91) |  | 3.72 (0.46-30.39) |  |
|  | Dominant | AA | 316 (75.8) | 114 (72.2) | 1 | 0.370 | 1 | 0.430 |
|  |  | AG/GG | 101 (24.2) | 44 (27.9) | 0.83 (0.55-1.25) |  | 0.84 (0.55-1.29) |  |
|  | Recessive | AA/AG | 408 (97.8) | 157 (99.4) | 1 | 0.170 | 1 | 0.130 |
|  |  | GG | 9 (2.2) | 1 (0.6) | 3.46 (0.44-27.56) |  | 3.96 (0.49-32.33) |  |
| rs135538 | Allele | G | 464 (55.4) | 180 (57.0) |  | 0.627 |  |  |
|  |  | C | 374 (44.6) | 136 (43.0) |  |  |  |  |
|  | Codominant | GG | 134 (32.0) | 54 (34.2) | 1 | 0.880 | 1 | 0.720 |
|  |  | GC | 196 (46.8) | 72 (45.6) | 1.10 (0.72-1.66) |  | 1.13 (0.74-1.73) |  |
|  |  | CC | 89 (21.2) | 32 (20.2) | 1.12 (0.67-1.87) |  | 1.23 (0.73-2.10) |  |
|  | Dominant | GG | 134 (32.0) | 54 (34.2) | 1 | 0.620 | 1 | 0.470 |
|  |  | GC/CC | 285 (68.0) | 104 (65.8) | 1.10 (0.75-1.63) |  | 1.16 (0.78-1.73) |  |
|  | Recessive | GG/GC | 330 (78.8) | 126 (79.8) | 1 | 0.790 | 1 | 0.560 |
|  |  | CC | 89 (21.2) | 32 (20.2) | 1.06 (0.67-1.67) |  | 1.15 (0.72-1.83) |  |
| rs4253681 | Allele | T | 679 (80.8) | 253 (79.6) |  | 0.626 |  |  |
|  |  | C | 161 (19.2) | 65 (20.4) |  |  |  |  |
|  | Codominant | TT | 276 (65.7) | 98 (61.6) | 1 | 0.330 | 1 | 0.230 |
|  |  | TC | 127 (30.2) | 57 (35.9) | 0.79 (0.54-1.17) |  | 0.77 (0.51-1.14) |  |
|  |  | CC | 17 (4.0) | 4 (2.5) | 1.51 (0.50-4.59) |  | 1.72 (0.54-5.42) |  |
|  | Dominant | TT | 276 (65.7) | 98 (61.6) | 1 | 0.360 | 1 | 0.330 |
|  |  | TC/CC | 144 (34.3) | 61 (38.4) | 0.84 (0.57-1.22) |  | 0.83 (0.56-1.22) |  |
|  | Recessive | TT/TC | 403 (96.0) | 155 (97.5) | 1 | 0.360 | 1 | 0.260 |
|  |  | CC | 17 (40) | 4 (2.5) | 1.63 (0.54-4.93) |  | 1.87 (0.60-5.86) |  |
| rs4253747 | Allele | T | 662 (78.6) | 260 (81.8) |  | 0.238 |  |  |
|  |  | A | 180 (21.4) | 58 (18.2) |  |  |  |  |
|  | Codominant | TT | 262 (62.2) | 105 (66.0) | 1 | 0.340 | 1 | 0.410 |
|  |  | TA | 138 (32.8) | 50 (31.4) | 1.11 (0.75-1.64) |  | 1.10 (0.73-1.65) |  |
|  |  | AA | 21 (5.0) | 4 (2.5) | 2.10 (0.71-6.28) |  | 2.02 (0.65-6.27) |  |
|  | Dominant | TT | 262 (62.2) | 105 (66.0) | 1 | 0.390 | 1 | 0.440 |
|  |  | TA/AA | 159 (37.8) | 54 (34.0) | 1.18 (0.80-1.73) |  | 1.17 (0.79-1.73) |  |
|  | Recessive | TT/TA | 400 (95.0) | 155 (97.5) | 1 | 0.170 | 1 | 0.210 |
|  |  | AA | 21 (5.0) | 4 (2.5) | 2.03 (0.69-6.02) |  | 1.96 (0.64-6.04) |  |
| rs2009873 | Allele | A | 478 (57.0) | 188 (59.9) |  | 0.386 |  |  |
|  |  | G | 360 (43.0) | 126 (40.1) |  |  |  |  |
|  | Codominant | AA | 137 (32.7) | 59 (37.6) | 1 | 0.540 | 1 | 0.380 |
|  |  | AG | 204 (48.7) | 70 (44.6) | 1.26 (0.83-1.89) |  | 1.35 (0.89-2.05) |  |
|  |  | GG | 78 (18.6) | 28 (17.8) | 1.20 (0.71-2.04) |  | 1.17 (0.68-2.01) |  |
|  | Dominant | AA | 137 (32.7) | 59 (37.6) | 1 | 0.270 | 1 | 0.200 |
|  |  | AG/GG | 282 (67.3) | 98 (62.4) | 1.24 (0.85-1.82) |  | 1.30 (0.88-1.92) |  |
|  | Recessive | AA/AG | 341 (81.4) | 129 (82.2) | 1 | 0.830 | 1 | 0.960 |
|  |  | GG | 78 (18.6) | 28 (17.8) | 1.05 (0.65-1.70) |  | 0.99 (0.60-1.61) |  |
| rs2066140 | Allele | G | 476 (56.7) | 191 (60.4) |  | 0.247 |  |  |
|  |  | C | 364 (43.3) | 125 (39.6) |  |  |  |  |
|  | Codominant | GG | 134 (31.9) | 60 (38.0) | 1 | 0.390 | 1 | 0.310 |
|  |  | GC | 208 (49.5) | 71 (44.9) | 1.31 (0.87-1.97) |  | 1.39 (0.91-2.11) |  |
|  |  | CC | 78 (18.6) | 27 (17.1) | 1.29 (0.76-2.20) |  | 1.26 (0.73-2.17) |  |
|  | Dominant | GG | 134 (31.9) | 60 (38.0) | 1 | 0.170 | 1 | 0.140 |
|  |  | GC/CC | 286 (68.1) | 98 (62.0) | 1.31 (0.89-1.91) |  | 1.35 (0.91-2.00) |  |
|  | Recessive | GG/GC | 342 (81.4) | 131 (82.9) | 1 | 0.680 | 1 | 0.870 |
|  |  | CC | 78 (18.6) | 27 (17.1) | 1.11 (0.68-1.79) |  | 1.04 (0.63-1.72) |  |
| rs2739513 | Allele | A | 470 (57.2) | 182 (59.9) |  | 0.417 |  |  |
|  |  | G | 352 (42.8) | 122 (40.1) |  |  |  |  |
|  | Codominant | AA | 135 (32.9) | 57 (37.5) | 1 | 0.580 | 1 | 0.420 |
|  |  | AG | 200 (48.7) | 68 (44.7) | 1.24 (0.82-1.88) |  | 1.33 (0.87-2.04) |  |
|  |  | GG | 76 (18.5) | 27 (17.8) | 1.19 (0.69-2.03) |  | 1.16 (0.67-2.02) |  |
|  | Dominant | AA | 135 (32.9) | 57 (37.5) | 1 | 0.300 | 1 | 0.220 |
|  |  | AG/GG | 276 (67.2) | 95 (62.5) | 1.23 (0.83-1.81) |  | 1.28 (0.86-1.91) |  |
|  | Recessive | AA/AG | 335 (81.5) | 125 (82.2) | 1 | 0.840 | 1 | 0.960 |
|  |  | GG | 76 (18.5) | 27 (17.8) | 1.05 (0.65-1.71) |  | 0.99 (0.60-1.63) |  |
| rs2486736 | Allele | A | 470 (56.5) | 189 (60.6) |  | 0.213 |  |  |
|  |  | G | 362 (43.5) | 123 (39.4) |  |  |  |  |
|  | Codominant | AA | 131 (31.5) | 59 (37.8) | 1 | 0.360 | 1 | 0.270 |
|  |  | AG | 208 (50.0) | 71 (45.5) | 1.32 (0.88-1.99) |  | 1.41 (0.92-2.15) |  |
|  |  | GG | 77 (18.5) | 26 (16.7) | 1.33 (0.78-2.29) |  | 1.30 (0.74-2.27) |  |
|  | Dominant | AA | 131 (31.5) | 59 (37.8) | 1 | 0.150 | 1 | 0.110 |
|  |  | AG/GG | 285 (68.5) | 97 (62.2) | 1.32 (0.90-1.94) |  | 1.38 (0.93-2.05) |  |
|  | Recessive | AA/AG | 339 (81.5) | 130 (83.3) | 1 | 0.610 | 1 | 0.800 |
|  |  | GG | 77 (18.5) | 26 (16.7) | 1.14 (0.70-1.85) |  | 1.07 (0.64-1.77) |  |
| rs480902 | Allele | C | 477 (56.8) | 191 (60.1) |  | 0.314 |  |  |
|  |  | T | 363 (43.2) | 127 (39.9) |  |  |  |  |
|  | Codominant | CC | 134 (31.9) | 60 (37.7) | 1 | 0.400 | 1 | 0.300 |
|  |  | CT | 209 (49.8) | 71 (44.6) | 1.32 (0.88-1.98) |  | 1.39 (0.92-2.12) |  |
|  |  | TT | 77 (18.3) | 28 (17.6) | 1.23 (0.73-2.09) |  | 1.20 (0.69-2.06) |  |
|  | Dominant | CC | 134 (31.9) | 60 (37.7) | 1 | 0.190 | 1 | 0.150 |
|  |  | CT/TT | 286 (68.1) | 99 (62.3) | 1.29 (0.88-1.89) |  | 1.34 (0.90-1.98) |  |
|  | Recessive | CC/CT | 343 (81.7) | 131 (82.4) | 1 | 0.840 | 1 | 0.970 |
|  |  | TT | 77 (18.3) | 28 (17.6) | 1.05 (0.65-1.69) |  | 0.99 (0.61-1.62) |  |
| rs508618 | Allele | A | 748 (89.0) | 282 (89.2) |  | 0.925 |  |  |
|  |  | G | 92 (11.0) | 34 (10.8) |  |  |  |  |
|  | Codominant | AA | 331 (78.8) | 124 (78.5) | 1 | 0.370 | 1 | 0.260 |
|  |  | AG | 86 (20.5) | 34 (21.5) | 0.95 (0.61-1.48) |  | 0.98 (0.62-1.55) |  |
|  |  | GG | 3 (0.7) | 0 (0.0) | NA (0.00-NA) |  | NA (0.00-NA) |  |
|  | Dominant | AA | 331 (78.8) | 124 (78.5) | 1 | 0.930 | 1 | 0.930 |
|  |  | AG/GG | 89 (21.2) | 34 (21.5) | 0.98 (0.63-1.53) |  | 1.02 (0.64-1.61) |  |
|  | Recessive | AA/AG | 417 (99.3) | 158 (100.0) | 1 | 0.170 | 1 | 0.100 |
|  |  | GG | 3 (0.7) | 0 (0.0) | NA (0.00-NA) |  | NA (0.00-NA) |  |
| rs2790882 | Allele | A | 471 (56.5) | 189 (59.8) |  | 0.307 |  |  |
|  |  | G | 363 (43.5) | 127 (40.2) |  |  |  |  |
|  | Codominant | AA | 131 (31.4) | 59 (37.3) | 1 | 0.390 | 1 | 0.270 |
|  |  | AG | 209 (50.1) | 71 (44.9) | 1.33 (0.88-1.99) |  | 1.41 (0.93-2.15) |  |
|  |  | GG | 77 (18.5) | 28 (17.7) | 1.24 (0.73-2.11) |  | 1.21 (0.70-2.09) |  |
|  | Dominant | AA | 131 (31.4) | 59 (37.3) | 1 | 0.180 | 1 | 0.130 |
|  |  | AG/GG | 286 (68.6) | 99 (62.7) | 1.30 (0.89-1.91) |  | 1.35 (0.91-2.01) |  |
|  | Recessive | AA/AG | 340 (81.5) | 130 (82.3) | 1 | 0.840 | 1 | 0.970 |
|  |  | GG | 77 (18.5) | 28 (17.7) | 1.05 (0.65-1.69) |  | 0.99 (0.61-1.62) |  |
| rs2486729 | Allele | A | 466 (55.9) | 190 (59.7) |  | 0.235 |  |  |
|  |  | G | 368 (44.1) | 128 (40.3) |  |  |  |  |
|  | Codominant | AA | 137 (32.9) | 60 (37.7) | 1 | 0.510 | 1 | 0.460 |
|  |  | AG | 192 (46.0) | 70 (44.0) | 1.20 (0.80-1.81) |  | 1.28 (0.84-1.95) |  |
|  |  | GG | 88 (21.1) | 29 (18.2) | 1.33 (0.79-2.23) |  | 1.30 (0.76-2.21) |  |
|  | Dominant | AA | 137 (32.9) | 60 (37.7) | 1 | 0.270 | 1 | 0.210 |
|  |  | AG/GG | 280 (67.2) | 99 (62.3) | 1.24 (0.85-1.81) |  | 1.28 (0.87-1.90) |  |
|  | Recessive | AA/AG | 329 (78.9) | 130 (81.8) | 1 | 0.440 | 1 | 0.610 |
|  |  | GG | 88 (21.1) | 29 (18.2) | 1.20 (0.75-1.91) |  | 1.13 (0.70-1.83) |  |
| rs7542797 | Allele | A | 729 (87.0) | 281 (88.9) |  | 0.376 |  |  |
|  |  | C | 109 (13.0) | 35 (11.1) |  |  |  |  |
|  | Codominant | AA | 317 (75.7) | 124 (78.5) | 1 | 0.510 | 1 | 0.610 |
|  |  | AC | 95 (22.7) | 33 (20.9) | 1.13 (0.72-1.76) |  | 1.10 (0.70-1.75) |  |
|  |  | CC | 7 (1.7) | 1 (0.6) | 2.74 (0.33-22.49) |  | 2.52 (0.30-21.47) |  |
|  | Dominant | AA | 317 (75.7) | 124 (78.5) | 1 | 0.470 | 1 | 0.560 |
|  |  | AC/CC | 102 (24.3) | 34 (21.5) | 1.17 (0.76-1.82) |  | 1.14 (0.73-1.80) |  |
|  | Recessive | AA/AC | 412 (98.3) | 157 (99.4) | 1 | 0.300 | 1 | 0.360 |
|  |  | CC | 7 (1.7) | 1 (0.6) | 2.67 (0.33-21.86) |  | 2.46 (0.29-20.89) |  |
| rs1339891 | Allele | G | 755 (89.9) | 289 (90.9) |  | 0.610 |  |  |
|  |  | A | 85 (10.1) | 29 (9.1) |  |  |  |  |
|  | Codominant | GG | 340 (81.0) | 130 (81.8) | 1 | 0.200 | 1 | 0.270 |
|  |  | GA | 75 (17.9) | 29 (18.2) | 0.99 (0.62-1.59) |  | 0.95 (0.58-1.54) |  |
|  |  | AA | 5 (1.2) | 0 (0.0) | NA (0.00-NA) |  | NA (0.00-NA) |  |
|  | Dominant | GG | 340 (81.0) | 130 (81.8) | 1 | 0.820 | 1 | 0.990 |
|  |  | GA/AA | 80 (19.1) | 29 (18.2) | 1.05 (0.66-1.69) |  | 1.00 (0.62-1.63) |  |
|  | Recessive | GG/GA | 415 (98.8) | 159 (100.0) | 1 | 0.072 | 1 | 0.110 |
|  |  | AA | 5 (1.2) | 0 (0.0) | NA (0.00-NA) |  | NA (0.00-NA) |  |
| rs12406290 | Allele | A | 426 (52.3) | 167 (54.2) |  | 0.572 |  |  |
|  |  | G | 388 (47.7) | 141 (45.8) |  |  |  |  |
|  | Codominant | AA | 111 (27.3) | 47 (30.5) | 1 | 0.740 | 1 | 0.700 |
|  |  | AG | 204 (50.1) | 73 (47.4) | 1.18 (0.77-1.83) |  | 1.21 (0.77-1.89) |  |
|  |  | GG | 92 (22.6) | 34 (22.1) | 1.15 (0.68-1.93) |  | 1.15 (0.67-1.97) |  |
|  | Dominant | AA | 111 (27.3) | 47 (30.5) | 1 | 0.450 | 1 | 0.420 |
|  |  | AG/GG | 296 (72.7) | 107 (69.5) | 1.17 (0.78-1.76) |  | 1.19 (0.78-1.81) |  |
|  | Recessive | AA/AG | 315 (77.4) | 120 (77.9) | 1 | 0.890 | 1 | 0.940 |
|  |  | GG | 92 (22.6) | 34 (22.1) | 1.03 (0.66-1.61) |  | 1.02 (0.64-1.61) |  |
| rs2153364 | Allele | A | 396 (51.4) | 156 (53.8) |  | 0.492 |  |  |
|  |  | G | 374 (48.6) | 134 (46.2) |  |  |  |  |
|  | Codominant | AA | 102 (26.5) | 44 (30.3) | 1 | 0.680 | 1 | 0.720 |
|  |  | AG | 192 (49.9) | 68 (46.9) | 1.22 (0.78-1.91) |  | 1.21 (0.76-1.91) |  |
|  |  | GG | 91 (23.6) | 33 (22.8) | 1.19 (0.70-2.03) |  | 1.17 (0.67-2.02) |  |
|  | Dominant | AA | 102 (26.5) | 44 (30.3) | 1 | 0.380 | 1 | 0.430 |
|  |  | AG/GG | 283 (73.5) | 101 (69.7) | 1.21 (0.79-1.84) |  | 1.19 (0.77-1.84) |  |
|  | Recessive | AA/AG | 294 (76.4) | 112 (77.2) | 1 | 0.830 | 1 | 0.880 |
|  |  | GG | 91 (23.6) | 33 (22.8) | 1.05 (0.67-1.65) |  | 1.04 (0.65-1.65) |  |
| rs2275279 | Allele | A | 616 (73.3) | 225 (70.8) |  | 0.380 |  |  |
|  |  | T | 224 (26.7) | 93 (29.2) |  |  |  |  |
|  | Codominant | AA | 231 (55.0) | 80 (50.3) | 1 | 0.590 | 1 | 0.700 |
|  |  | AT | 154 (36.7) | 65 (40.9) | 0.82 (0.56-1.21) |  | 0.84 (0.57-1.25) |  |
|  |  | TT | 35 (8.3) | 14 (8.8) | 0.87 (0.44-1.69) |  | 0.89 (0.45-1.78) |  |
|  | Dominant | AA | 231 (55.0) | 80 (50.3) | 1 | 0.310 | 1 | 0.410 |
|  |  | AT/TT | 189 (45.0) | 79 (49.7) | 0.83 (0.57-1.19) |  | 0.85 (0.59-1.24) |  |
|  | Recessive | AA/AT | 385 (91.7) | 145 (91.2) | 1 | 0.860 | 1 | 0.910 |
|  |  | TT | 35 (8.3) | 14 (8.8) | 0.94 (0.49-1.80) |  | 0.96 (0.49-1.87) |  |
| rs2301104 | Allele | G | 786 (93.6) | 296 (93.1) |  | 0.764 |  |  |
|  |  | C | 54 (6.4) | 22 (6.9) |  |  |  |  |
|  | Codominant | GG | 366 (87.1) | 138 (86.8) | 1 | 0.270 | 1 | 0.260 |
|  |  | GC | 54 (12.9) | 20 (12.6) | 1.02 (0.59-1.76) |  | 0.98 (0.56-1.73) |  |
|  |  | CC | 0 (0.0) | 1 (0.6) | 0.00 (0.00-NA) |  | 0.00 (0.00-NA) |  |
|  | Dominant | GG | 366 (87.1) | 138 (86.8) | 1 | 0.910 | 1 | 0.810 |
|  |  | GC/CC | 54 (12.9) | 21 (13.2) | 0.97 (0.56-1.67) |  | 0.93 (0.53-1.63) |  |
|  | Recessive | GG/GC | 420 (100.0) | 158 (99.4) | 1 | 0.110 | 1 | 0.100 |
|  |  | CC | 0 (0.0) | 1 (0.6) | 0.00 (0.00-NA) |  | 0.00 (0.00-NA) |  |
| rs12434438 | Allele | A | 626 (76.2) | 239 (76.1) |  | 0.988 |  |  |
|  |  | G | 196 (23.8) | 75 (23.9) |  |  |  |  |
|  | Codominant | AA | 230 (56.0) | 93 (59.2) | 1 | 0.130 | 1 | 0.090 |
|  |  | AG | 166 (40.4) | 53 (33.8) | 1.27 (0.86-1.87) |  | 1.36 (0.91-2.04) |  |
|  |  | GG | 15 (3.6) | 11 (7.0) | 0.55 (0.24-1.25) |  | 0.57 (0.25-1.32) |  |
|  | Dominant | AA | 230 (56.0) | 93 (59.2) | 1 | 0.480 | 1 | 0.310 |
|  |  | AG/GG | 181 (44.0) | 64 (40.8) | 1.14 (0.79-1.66) |  | 1.22 (0.83-1.80) |  |
|  | Recessive | AA/AG | 396 (96.3) | 146 (93.0) | 1 | 0.100 | 1 | 0.110 |
|  |  | GG | 15 (3.6) | 11 (7.0) | 0.50 (0.23-1.12) |  | 0.50 (0.22-1.14) |  |
| rs966824 | Allele | C | 692 (82.8) | 256 (82.1) |  | 0.774 |  |  |
|  |  | T | 144 (17.2) | 56 (17.9) |  |  |  |  |
|  | Codominant | CC | 282 (67.5) | 106 (68.0) | 1 | 0.400 | 1 | 0.400 |
|  |  | CT | 128 (30.6) | 44 (28.2) | 1.09 (0.73-1.65) |  | 1.14 (0.75-1.74) |  |
|  |  | TT | 8 (1.9) | 6 (3.8) | 0.50 (0.17-1.48) |  | 0.52 (0.17-1.59) |  |
|  | Dominant | CC | 282 (67.5) | 106 (68.0) | 1 | 0.910 | 1 | 0.740 |
|  |  | CT/TT | 136 (32.5) | 50 (32.0) | 1.02 (0.69-1.52) |  | 1.07 (0.71-1.60) |  |
|  | Recessive | CC/CT | 410 (98.1) | 150 (96.2) | 1 | 0.200 | 1 | 0.230 |
|  |  | TT | 8 (1.9) | 6 (3.8) | 0.49 (0.17-1.43) |  | 0.50 (0.16-1.51) |  |
| rs2301112 | Allele | A | 758 (95.7) | 282 (94.6) |  | 0.449 |  |  |
|  |  | C | 34 (4.3) | 16 (5.4) |  |  |  |  |
|  | Codominant | AA | 363 (91.7) | 133 (89.3) | 1 | 0.460 | 1 | 0.410 |
|  |  | AC | 32 (8.1) | 16 (10.7) | 0.73 (0.39-1.38) |  | 0.67 (0.35-1.28) |  |
|  |  | CC | 1 (0.2) | 0 (0.0) | NA (0.00-NA) |  | NA (0.00-NA) |  |
|  | Dominant | AA | 363 (91.7) | 133 (89.3) | 1 | 0.390 | 1 | 0.260 |
|  |  | AC/CC | 33 (8.3) | 16 (10.7) | 0.76 (0.40-1.42) |  | 0.68 (0.36-1.31) |  |
|  | Recessive | AA/AC | 395 (99.8) | 149 (100.0) | 1 | 0.420 | 1 | 0.540 |
|  |  | CC | 1 (0.2) | 0 (0.0) | NA (0.00-NA) |  | NA (0.00-NA) |  |
| rs2301113 | Allele | A | 562 (67.4) | 204 (64.2) |  | 0.298 |  |  |
|  |  | C | 272 (32.6) | 114 (35.8) |  |  |  |  |
|  | Codominant | AA | 180 (43.2) | 70 (44.0) | 1 | 0.027* | 1 | 0.083 |
|  |  | AC | 202 (48.4) | 64 (40.3) | 1.23 (0.83-1.82) |  | 1.17 (0.78-1.75) |  |
|  |  | CC | 35 (8.4) | 25 (15.7) | 0.54 (0.30-0.98) |  | 0.58 (0.32-1.06) |  |
|  | Dominant | AA | 180 (43.2) | 70 (44.0) | 1 | 0.850 | 1 | 0.960 |
|  |  | AC/CC | 237 (56.8) | 89 (56.0) | 1.04 (0.72-1.50) |  | 1.01 (0.69-1.48) |  |
|  | Recessive | AA/AC | 382 (91.6) | 134 (84.3) | 1 | 0.013* | 1 | 0.036* |
|  |  | CC | 35 (8.4) | 25 (15.7) | 0.49 (0.28-0.85) |  | 0.54 (0.30-0.95) |  |
| rs2295778 | Allele | C | 643 (76.5) | 235 (74.4) |  | 0.439 |  |  |
|  |  | G | 197 (23.5) | 81 (25.6) |  |  |  |  |
|  | Codominant | CC | 245 (58.3) | 89 (56.3) | 1 | 0.570 | 1 | 0.660 |
|  |  | CG | 153 (36.4) | 57 (36.1) | 0.98 (0.66-1.44) |  | 1.04 (0.69-1.55) |  |
|  |  | GG | 22 (5.2) | 12 (7.6) | 0.67 (0.32-1.40) |  | 0.72 (0.33-1.54) |  |
|  | Dominant | CC | 245 (58.3) | 89 (56.3) | 1 | 0.660 | 1 | 0.910 |
|  |  | CG/GG | 175 (41.7) | 69 (43.7) | 0.92 (0.64-1.33) |  | 0.98 (0.67-1.43) |  |
|  | Recessive | CC/CG | 398 (94.8) | 146 (92.4) | 1 | 0.300 | 1 | 0.370 |
|  |  | GG | 22 (5.2) | 12 (7.6) | 0.67 (0.32-1.39) |  | 0.71 (0.33-1.49) |  |
| rs11190602 | Allele | T | 746 (88.8) | 275 (87.0) |  | 0.400 |  |  |
|  |  | C | 94 (11.2) | 41 (13.0) |  |  |  |  |
|  | Codominant | TT | 332 (79.0) | 121 (76.6) | 1 | 0.620 | 1 | 0.500 |
|  |  | TC | 82 (19.5) | 33 (20.9) | 0.91 (0.57-1.43) |  | 0.88 (0.55-1.41) |  |
|  |  | CC | 6 (1.4) | 4 (2.5) | 0.55 (0.15-1.97) |  | 0.47 (0.13-1.78) |  |
|  | Dominant | TT | 332 (79.0) | 121 (76.6) | 1 | 0.520 | 1 | 0.430 |
|  |  | TC/CC | 88 (20.9) | 37 (23.4) | 0.87 (0.56-1.34) |  | 0.83 (0.53-1.31) |  |
|  | Recessive | TT/TC | 414 (98.6) | 154 (97.5) | 1 | 0.380 | 1 | 0.300 |
|  |  | CC | 6 (1.4) | 4 (2.5) | 0.56 (0.16-2.00) |  | 0.49 (0.13-1.82) |  |
| rs3750633 | Allele | G | 778 (92.6) | 289 (90.9) |  | 0.326 |  |  |
|  |  | A | 62 (7.4) | 29 (9.1) |  |  |  |  |
|  | Codominant | GG | 359 (85.5) | 134 (84.3) | 1 | 0.048* | 1 | 0.064 |
|  |  | GA | 60 (14.3) | 21 (13.2) | 1.07 (0.62-1.82) |  | 1.02 (0.58-1.77) |  |
|  |  | AA | 1 (0.2) | 4 (2.5) | 0.09 (0.01-0.84) |  | 0.10 (0.01-0.92) |  |
|  | Dominant | GG | 359 (85.5) | 134 (84.3) | 1 | 0.720 | 1 | 0.600 |
|  |  | GA/AA | 61 (14.5) | 25 (15.7) | 0.91 (0.55-1.51) |  | 0.87 (0.51-1.47) |  |
|  | Recessive | GG/GA | 419 (99.8) | 155 (97.5) | 1 | 0.014* | 1 | 0.019* |
|  |  | AA | 1 (0.2) | 4 (2.5) | 0.09 (0.01-0.83) |  | 0.10 (0.01-0.92) |  |
| rs10883512 | Allele | A | 781 (92.8) | 289 (91.5) |  | 0.457 |  |  |
|  |  | G | 61 (7.2) | 27 (8.5) |  |  |  |  |
|  | Codominant | AA | 361 (85.8) | 134 (84.8) | 1 | 0.140 | 1 | 0.180 |
|  |  | AG | 59 (14) | 21 (13.3) | 1.04 (0.61-1.78) |  | 1.01 (0.58-1.75) |  |
|  |  | GG | 1 (0.2) | 3 (1.9) | 0.12 (0.01-1.20) |  | 0.14 (0.01-1.39) |  |
|  | Dominant | AA | 361 (85.8) | 134 (84.8) | 1 | 0.780 | 1 | 0.680 |
|  |  | AG/GG | 60 (14.2) | 24 (15.2) | 0.93 (0.56-1.55) |  | 0.90 (0.53-1.52) |  |
|  | Recessive | AA/AG | 420 (99.8) | 155 (98.1) | 1 | 0.046 | 1 | 0.066 |
|  |  | GG | 1 (0.2) | 3 (1.9) | 0.12 (0.01-1.19) |  | 0.14 (0.01-1.39) |  |
| rs11816840 | Allele | G | 779 (92.5) | 289 (91.5) |  | 0.548 |  |  |
|  |  | C | 63 (7.5) | 27 (8.5) |  |  |  |  |
|  | Codominant | GG | 359 (85.3) | 134 (84.8) | 1 | 0.130 | 1 | 0.180 |
|  |  | GC | 61 (14.5) | 21 (13.3) | 1.08 (0.64-1.85) |  | 1.04 (0.60-1.81) |  |
|  |  | CC | 1 (0.2) | 3 (1.9) | 0.12 (0.01-1.21) |  | 0.14 (0.01-1.40) |  |
|  | Dominant | GG | 359 (85.3) | 134 (84.8) | 1 | 0.890 | 1 | 0.780 |
|  |  | GC/CC | 62 (14.7) | 24 (15.2) | 0.96 (0.58-1.61) |  | 0.93 (0.55-1.57) |  |
|  | Recessive | GG/GC | 420 (99.8) | 155 (98.1) | 1 | 0.046 | 1 | 0.066 |
|  |  | CC | 1 (0.2) | 3 (1.9) | 0.12 (0.01-1.19) |  | 0.14 (0.01-1.39) |  |
| rs1054399 | Allele | C | 778 (92.6) | 289 (90.9) |  | 0.326 |  |  |
|  |  | T | 62 (7.4) | 29 (9.1) |  |  |  |  |
|  | Codominant | CC | 359 (85.5) | 134 (84.3) | 1 | 0.048* | 1 | 0.064 |
|  |  | CT | 60 (14.3) | 21 (13.2) | 1.07 (0.62-1.82) |  | 1.02 (0.58-1.77) |  |
|  |  | TT | 1 (0.2) | 4 (2.5) | 0.09 (0.01-0.84) |  | 0.10 (0.01-0.92) |  |
|  | Dominant | CC | 359 (85.5) | 134 (84.3) | 1 | 0.720 | 1 | 0.600 |
|  |  | CT/TT | 61 (14.5) | 25 (15.7) | 0.91 (0.55-1.51) |  | 0.87 (0.51-1.47) |  |
|  | Recessive | CC/CT | 419 (99.8) | 155 (97.5) | 1 | 0.014* | 1 | 0.019* |
|  |  | TT | 1 (0.2) | 4 (2.5) | 0.09 (0.01-0.83) |  | 0.10 (0.01-0.92) |  |
| rs11292 | Allele | T | 779 (92.5) | 289 (90.9) |  | 0.357 |  |  |
|  |  | C | 63 (7.5) | 29 (9.1) |  |  |  |  |
|  | Codominant | TT | 359 (85.3) | 134 (84.3) | 1 | 0.046* | 1 | 0.063 |
|  |  | TC | 61 (14.5) | 21 (13.2) | 1.08 (0.64-1.85) |  | 1.04 (0.60-1.81) |  |
|  |  | CC | 1 (0.2) | 4 (2.5) | 0.09 (0.01-0.84) |  | 0.10 (0.01-0.92) |  |
|  | Dominant | TT | 359 (85.3) | 134 (84.3) | 1 | 0.770 | 1 | 0.660 |
|  |  | TC/CC | 62 (14.7) | 25 (15.7) | 0.93 (0.56-1.53) |  | 0.89 (0.53-1.50) |  |
|  | Recessive | TT/TC | 420 (99.8) | 155 (97.5) | 1 | 0.014* | 1 | 0.019* |
|  |  | CC | 1 (0.2) | 4 (2.5) | 0.09 (0.01-0.83) |  | 0.10 (0.01-0.91) |  |
| rs11190613 | Allele | T | 779 (92.5) | 289 (90.9) |  | 0.357 |  |  |
|  |  | C | 63 (7.5) | 29 (9.1) |  |  |  |  |
|  | Codominant | TT | 359 (85.3) | 134 (84.3) | 1 | 0.046* | 1 | 0.063 |
|  |  | TC | 61 (14.5) | 21 (13.2) | 1.08 (0.64-1.85) |  | 1.04 (0.60-1.81) |  |
|  |  | CC | 1 (0.2) | 4 (2.5) | 0.09 (0.01-0.84) |  | 0.10 (0.01-0.92) |  |
|  | Dominant | TT | 359 (85.3) | 134 (84.3) | 1 | 0.770 | 1 | 0.660 |
|  |  | TC/CC | 62 (14.7) | 25 (15.7) | 0.93 (0.56-1.53) |  | 0.89 (0.53-1.50) |  |
|  | Recessive | TT/TC | 420 (99.8) | 155 (97.5) | 1 | 0.014* | 1 | 0.019* |
|  |  | CC | 1 (0.2) | 4 (2.5) | 0.09 (0.01-0.83) |  | 0.10 (0.01-0.91) |  |

Allelic frequencies were compared by chi-square test (2*2 contingency table, df = 1). Association between SNPs and HAH under different models was detected by using binary logistic regression. **^a^** Adjusted for age, height, weight, BMI, smoking and drinking status, HR, SpO_2_, SBP and DBP. * P < 0.05 indicated statistical significance. HAH+, subjects with HAH; HAH-, subjects without HAH. See Table 3 for group abbreviations.
